# Editorial: Molecular links between metabolism and neural dysfunction

**DOI:** 10.3389/fnins.2023.1212939

**Published:** 2023-05-30

**Authors:** Ari Meerson, Or Shahar, Inbal Mor

**Affiliations:** ^1^Molecular Biology of Chronic Diseases Laboratory/Genomic Center, Galilee Research Institute (MIGAL), Kiryat Shmona, Israel; ^2^Faculty of Sciences, Tel Hai Academic College, Upper Galilee, Israel; ^3^Ruth and Bruce Rappaport Faculty of Medicine, Technion-Israel Institute of Technology, Haifa, Israel

**Keywords:** diet, obesity, nutrition, exercise, cognition, neurodegeneration, Alzheimer's

Chronic dysfunction of the nervous system, including autism spectrum disorders and anxiety disorders, is broadly correlated with metabolic dysfunction, such as obesity and diabetes (Farooqui et al., 2012; Nousen et al., 2013; Ogrodnik et al., 2019). Both neural and metabolic disorders are a worldwide epidemiological concern, and the link between them suggests common molecular mechanisms of pathogenesis, which warrant close attention. Additionally, changes in metabolic pathways or activity of specific enzymes have been suggested to play key roles in neuronal activity and brain function. Several mechanisms underlying this connection have been proposed, including chronic inflammation, mitochondrial dysfunction, and altered inter-tissue signaling by endocrine factors and by non-coding RNAs. Nevertheless, the exact mechanisms remain mostly unknown.

The medical community stresses the importance of an active lifestyle and healthy diet to maintain long-term health. In this Research Topic, Katz et al. examined the effect of physical exercise on metabolic regulation in the brain, specifically in the hippocampus, a brain region involved in learning and memory, and in the hypothalamus, the region regulating physiological homeostasis. The authors subjected mice to different types of long-term (4 weeks) exercise and examined the effects on the central molecular pathways that respond to metabolic stimuli, namely, AMPK and AKT and their downstream effector, the master regulator, mTOR, which coordinates energetic status and protein synthesis (Hawley et al., 2014). While both AMPK and AKT signaling responded to physical activity, the hypothalamus more closely recapitulated the pattern found in skeletal muscle during exercise. The hypothalamus–muscle response was characteristic of energetic deficits present in muscle following exercise. The different responses in specific brain regions point to the complex effect exercise has on the brain. Differential activation of molecular pathways that sense cellular energetics can underlie the effects different types of exercise have on brain function.

Obesity and hyperglycemia are associated with a higher incidence of cognitive decline and Alzheimer's disease (AD). There is evidence for cognitive benefits in patients with obesity following bariatric surgery (Nota et al., 2020). Interestingly, bariatric surgery has been shown to improve systemic metabolic parameters (such as blood glucose and cholesterol levels), independent of weight loss. Samuel et al. examined effects of bariatric surgery on AD. The authors investigated if lean mice in a model for AD could have cognitive benefits from sleeve gastrectomy (SG), thereby uncoupling the weight-loss-independent improvement that could stem from the surgery. The 5xFAD mice are transgenic for two human genes (APP and PSEN1), carrying several mutations associated with familial AD, and develop early-onset dementia. While SG did decrease blood glucose and cholesterol levels, it did not affect the number of β-amyloid plaques or cognition in 5xFAD mice. These results suggest that the main effect of bariatric surgery on cognitive function may be attributed to weight loss and improvement in the sequelae of obesity rather than metabolic or other outcomes of the surgery. It is important to consider that in this mouse model, β-amyloid plaques can develop at an early age before the surgery is performed, and reversing an already present condition may be hard to achieve. Additionally, this genetic model for AD may not recapitulate the etiology of the more common non-familial sporadic AD. This study therefore provides crucial insights into the risk the metabolic syndrome plays in AD development, demonstrating that in lean mice improving particular metabolic parameters by sleeve gastrectomy did not directly improve cognitive function.

Strath et al. examined the link between vitamin D levels and chronic pain. Vitamin D is classically known for its importance in calcium homeostasis and skeletal health, but it is also involved in other processes including diseases of the nervous system (Wrzosek et al., 2013). Patients with chronic pain display lower vitamin D levels, and the vitamin D receptor has been shown to interact with specific pain signaling pathways. In this study, the authors used brain imaging to investigate the correlation between vitamin D status and brain structures known to be involved in experiencing pain in patients with chronic knee pain. The patients' reports about their pain severity showed a continuous correlation with vitamin D levels, with the most significant difference between patients with insufficient and optimal vitamin D levels at both ends of the scale. White matter surface area and gray matter volume in regions associated with pain processing decreased in patients with lower vitamin D. While the direct link between brain structure sizes in these regions and pain experience and how vitamin D contributes to these parameters remain unclear, this offers an interesting mechanistic link. Furthermore, it sheds light on to the effect of nutrients such as vitamin D on chronic pain, pointing to potential strategies for improving pain management.

The link between brain dysfunction and metabolism is further evidenced by alterations in the metabolomic profile in the context of mental health problems in human subjects. Whipp et al. investigated associations between depression and 11 low-molecular-weight metabolites (amino acids and ketone bodies) in a longitudinal Finnish population-based twin cohort, with 725 blood plasma samples and accompanying self-reporting data collected in adolescence and young adulthood. Using high-throughput ^1^H nuclear magnetic resonance spectroscopy (NMR) to profile the metabolites, the study identified a significant negative association of plasma valine and leucine with depression. The same group previously identified ketone body 3-hydroxybutyrate as a biomarker of aggression (Whipp et al., 2021), highlighting the potential of metabolomic biomarkers in different types of mental health problems.

While the mechanisms linking metabolic and neural disorders remain mostly unknown, Chegodayev et al. proposed a hypothetical mechanism tying together a deficiency of ketone bodies with burst suppression in neurons and an abnormal electroencephalographic pattern, which is more prevalent in disorders characterized by corpus callosum abnormalities. Based on existing studies, the authors discussed several pathways through which ketone body metabolism could affect neuronal electrophysiology, including myelination, ATP-sensitive potassium channel signaling, and/or control of neurotransmitter release. The contribution of these mechanisms to the metabolic regulation of neuronal activity may present a potential target for therapeutic intervention and thus warrants further experimental studies.

Together, these studies exemplify and encourage an integrative view of metabolic and neural dysfunction and present recent advances in molecular studies highlighting this link.

## Author contributions

All authors listed have made a substantial, direct, and intellectual contribution to the work and approved it for publication.
